# *Lactobacillus casei* LC01 Regulates Intestinal Epithelial Permeability through miR-144 Targeting of OCLN and ZO1

**DOI:** 10.4014/jmb.2002.02059

**Published:** 2020-08-08

**Authors:** Qiuke Hou, Yongquan Huang, Yan Wang, Liu Liao, Zhaoyang Zhu, Wenjie Zhang, Yongshang Liu, Peiwu Li, Xinlin Chen, Fengbin Liu

**Affiliations:** 1 Department of Gastroenterology, The First Affiliated Hospital of Guangzhou University of Chinese Medicine, Guangzhou 510176, P.R. China; 2 School of Chinese Medicine, Hong Kong Baptist University, Hong Kong, China; 3 Department of Orthopedics, The Second Affiliated Hospital of Guangzhou University of Chinese Medicine, Guangzhou 510176, P.R. China; 4 Department of Preventive Medicine and Health Statistics, Guangzhou University of Chinese Medicine, Guangzhou 510176, Guangdong, P.R. China

**Keywords:** miR-144, intestinal epithelial permeability, lactobacilli, occludin (OCLN), zonula occludens 1 (ZO1/TJP1)

## Abstract

Our previous report determined that miR-144 is a key regulator of intestinal epithelial permeability in irritable bowel syndrome with diarrhea (IBS-D) rats. Recent evidence has shown that lactobacilli play an important role in the relief of IBS-D symptoms. However, few studies have addressed the mechanisms by which microRNAs and lactobacilli exert their beneficial effects on intestinal epithelial permeability. Hence, to elucidate whether miRNAs and lactobacilli play roles in intestinal epithelial barrier regulation, we compared miRNA expression levels in intestinal epithelial cells (IECs) under *Lactobacillus casei* (*L. casei* LC01) treatment. IECs and *L. casei* LC01 were co-cultured and then subjected to microRNA microarray assay. qRT-PCR, western blot and ELISA were used to detect the expression of occludin (OCLN) and zonula occludens 1 (ZO1/TJP1). The interaction between miRNAs and *L. casei* LC01 acting in IECs was investigated through transfection of RNA oligoribonucleotides and pcDNA 3.1 plasmid. The results are as follows: 1) *L. casei* LC01 decreased the expression of miR-144 and FD4 and promoted OCLN and ZO1 expression in IECs; 2) *L. casei* LC01 enhanced the barrier function of IECs via downregulation of miR-144 and upregulation of OCLN and ZO1; 3) Under *L. casei* LC01 treatment, OCLN and ZO1 overexpression could partially eliminate the promoting effect of miR-144 on intestinal permeability in IECs. Our results demonstrate that *L. casei* LC01 regulates intestinal permeability of IECs through miR-144 targeting of OCLN and ZO1. *L. casei* LC01 can be a possible therapeutic target for managing dysfunction of the intestinal epithelial barrier.

## Introduction

Irritable bowel syndrome [1] is a chronic, functional bowel disorder that is characterized by visceral pain and bloating and altered bowel habits in the absence of anatomical or biochemical abnormalities [2]. Up to 30% of IBS patients have been reported to develop acute gastroenteritis, and most are diagnosed with irritable bowel syndrome with diarrhea (IBS-D), which is a major subtype and correlates with changes in the intestinal microflora after infection [3]. Brain-gut axis dysfunction [4], visceral hypersensitivity [5], gastrointestinal dysmotility [6], and alterations in psychosomatic [7] or psychosocial behavior [8] have been implicated in the pathophysiology of IBS-D, but the exact mechanisms remain largely undefined.

Probiotics are beneficial bacteria that can improve health and maintain the micro-ecological balance of the host intestine. Screening probiotic strains with excellent probiotic characteristics has become a research hotspot. Studies have shown that the probiotic *Lactobacillus paracasei* LC01 (*L. casei* LC01) has good acid and bile salt resistance, and can tolerate a pH around 4.6 [9, 10]. L.casei LC01 has a strong bacteriostatic effect on *Streptococcus mutans* and can produce bacteriocin or bacteriocin-like polypeptides, usually class II bacteriocins [11]. This active polypeptide can have a significant bacteriostatic effect on gram-positive bacteria [12]. However, there is no report about the role of *L. casei* LC01 in IBS-D.

MicroRNAs (miRNAs) are a type of non-coding small-molecule RNA endogenously expressed in eukaryotic cell organisms and can act on the 3'-untranslated region (3'-UTR) of target gene mRNA [13]. If the miRNAs are completely complementary to the 3'-UTR, it may cause the target gene mRNA to degrade; if not completely complementary, it may inhibit the target gene mRNA translation [14]. Current research reports that miRNAs play an important role in regulating IBS-D intestinal epithelial permeability [15, 16]. We also found in our previous studies that the downregulation of miR-144 can target OCLN and ZO1 to reverse the intestinal epithelial permeability of IBS-D rats [17], but little is known about whether *L. casei* LC01 can regulate the expression of miR-144 in intestinal epithelial cells (IEC).

In this study, we aimed to evaluate whether and how *L. casei* LC01 affects miR-144 expression in IECs and regulates intestinal epithelial permeability, which may provide potential therapeutic strategies for the prevention and treatment of diseases related to intestinal mucosal barrier function.

## Materials and Methods

### Human IEC Lines

Human IEC lines (HCoEpiC, C1388) were purchased from the Chinese Academy of Sciences Shanghai Cell Bank and cultured in DMEM containing 100 ml/l fetal bovine serum in a CO_2_ incubator with saturated humidity at 37°C. Fibroblasts were removed using phase difference digestion and adherence. When 80%-90% of the IECs were adherent to culture plates, the IECs were passaged by trypsin digestion.

### *L. casei* LC01 Preparation

*L. casei* LC01 (No. 1.570) was purchased from the China General Microbiological Culture Collection Center and stored at a temperature below 25°C until use. *L. casei* LC01 were cultured and stocked as previously reported [18]. Briefly, *L. casei* LC01 were cultured and incubated in a stationary state at 37°C for 48 h with MRS medium and plated to count effective colony forming units (CFU). *L. casei* LC01 were measured and adjusted to 1 × 10^9^ CFU/ml before use.

### Paracellular Flux Measurements

Intestinal epithelial permeability as a surrogate marker of layer integrity was measured by using fluorescein isothiocyanate-dextran 4 kDa (FD4; Sigma-Aldrich, USA). The IECs were treated with or without *L. casei* LC01 for 3 days, then FD4 (2 mg/ml) was added to the cultures, and the fluorescein isothiocyanate intensity of the basolateral fluid was measured with a fluorescence reader (FLUOstar Omega; BMG Labtech, Germany). The optical density (OD) value of each well was measured at a wavelength of 450 nm. The FD4 concentration was calculated and was expressed in picomoles (pmol).

### miRNA Microarray Analysis

IECs treated with or without *L. casei* LC01 for 3 days were subjected to a microRNA microarray assay. The method was as described previously [17]. In brief, two groups of IECs were used for the miRNA microarray assay, which was performed at Guangzhou RiboBio Co., Ltd. The assay consisted of four steps: prehybridization, hybridization, hybridization washing, and imaging. The CustomArray microarray was assembled using a hybridization cap and clips, and then prehybridization was performed. The hybridization chambers were filled with nuclease-free water, incubated at 65°C for 10 min, and then gradually reduced to room temperature. After the water was drawn out of the hybridization chamber, the chamber was filled with prehybridization solution, incubated at 37°C for 60 min and gently rotated in the hybridization oven. Next, the hybridization was performed with the hybridization solution prepared according to the following procedure: The total RNA from the distal colon in the two groups was extracted using the TRIzol method and fluorescently labeled with Cy3 using the Universal Linkage System (ULS) protocol, and then the solution was denatured at 95°C for 3 min and cooled on ice for 20 s to prepare for the hybridization steps. The hybridization chambers were filled with hybridization solution, followed by removal of the prehybridization solution, gentle mixing and incubation at 37°C for 16 h. Finally, the microarray was washed to reduce the specific hybridization background, covered with the imaging solution, and loaded into the GenePix 4000B microarray scanner.

### Enzyme-Linked Immunosorbent Assays (ELISA)

The method was as described previously [17]. Briefly, the IECs were treated with or without *L. casei* LC01 for 3 days and then harvested. The contents of OCLN (CUSABIO, CSB-E17291r, China) and ZO1 (CUSABIO, CSB-E17287r) in the culture medium were then determined using the corresponding ELISA kit according to the manufacturer’s instructions. The measurements were repeated in triplicate and averaged. The OD values (450 nm) indicated the relative amount of OCLN and ZO1.

### RNA Oligoribonucleotide Synthesis and Transfection

The method was as described previously [17]. Briefly, miR-144 mimic and inhibitor, mimic control and inhibitor control and the short interference RNAs (siRNA) were used to repress ZO1 or OCLN (siZO1 or siOCLN), and siRNA negative control was prepared for transfection (RiboBio, China). IECs treated with or without *L. casei* LC01 were seeded into 6-well plates, and once cell confluence reached 70%, cells were transfected with 100 nM RNA oligoribonucleotides (miR-144 mimic, miR-144 inhibitor, miR control, siRNA control, si-OCLN, or si-ZO1) in 2 ml serum-free reaction containing lipofectamine 2000 reagent for 6 h. IECs, without *L. casei* LC01 treatment, transfected with no RNA oligoribonucleotides were treated as the normal control group (NC), while IECs, without *L. casei* LC01 treatment, transfected with RNA control oligoribonucleotides were treated as negative control group. The IECs were then collected and examined using qRT-PCR and western blot. The sequences for cell transfection were as follows: miR-144 mimic: 5'-UACAGUAUAGAUGAUGUACU-3' (100 nM), miR-144 inhibitor: 5'- AUGUCAUAUCUACUACAUGA-3' (100 nM), miR control: 5'-UCGCAUGCCACAGCCAUGGC-3' (100 nM), siRNA control: 5'-UUCUCCGAACGUGUCACGUTT-3' (100 nM), si-ZO1: 5'-GUUAUACGAGCG AUCUCAU-3' (100 nM), si-OCLN: 5'-GGUUCUGGUGUGAACUAAATT-3' (100 nM).

### Quantitative Real-Time Polymerase Chain Reaction (qRT-PCR)

The method was as described previously [17]. Briefly, after total RNA was extracted and complementary DNA (cDNA) was synthesized, qRT-PCR was performed using an ABI Prism 7500 PCR system (Applied Biosystems, USA). β-Actin was used as an internal reference, and the 2^−ΔCt^ method was used to calculate the relative mRNA expression level. The measurements were repeated in triplicate and averaged. The primers were designed as follows: OCLN: 5’-AAGACGATGAGGTGCAGAAG-3’ (forward), 5’-GTGAAGAGAGCCTGACCAAA-3’ (reverse); ZO1: 5’-GGAGAGGTGTTTCGTGTTGT-3’ (forward), 5’-ACTGCTCAGCCCTGTTCTTA-3’(reverse); β-actin: 5’-CGTGACATTAAGGAGAAGCTG-3’(forward), 5’-CTAGAAGCATTTGCGGTGGAC-3’ (reverse); miR-144: 5'-GCGCGCTACAGTATAGATGATG-3' (forward), 5'-GCTGTCAACGATACGCTACG-3' (reverse).

### Western Blot

The method was as described previously [17]. Briefly, after proteins were extracted from the IECs, protein concentrations were determined using the BCA Protein Assay Kit (Beyotime, Beijing, China). Equal amounts of protein were loaded onto sodium dodecyl sulphate-polyacrylamide gels (SDS-PAGE) and transferred to polyvinylidene difluoride (PVDF) membranes. The membranes were incubated with primary antibodies (1:300 dilution) overnight at 4°C, incubated with a secondary antibody (1:3,000 dilution) at 37°C for 1 h, and examined using a chemiluminescence detection kit. β-Actin was used as the internal reference.

### Rescue Assay

The method was as described previously [17]. Briefly, IECs were transfected with the pcDNA 3.1 plasmid (pcDNA3.1-OCLN/ZO1), or the miR-144 mimic, or co-transfected with the overexpression vector and the miR-144 mimic. Western blot and qRT-PCR were then used to detect the expression of OCLN/ZO1.

### Statistical Analyses

SPSS 17.0 (SPSS Inc., USA) was used for statistical analyses. The measurement data that conformed to a normal distribution were expressed as the mean ± standard deviation. One-way analysis of variance (ANOVA) and least significant difference (LSD) multiple comparison tests were used to compare the differences between groups. Parameters with abnormal distribution were expressed as the median (quartile). Non-parametric (Mann-Whitney U) tests were used to compare the differences between groups. Differences were regarded as statistically significant when *p* < 0.05.

## Results

### *L. casei* LC01 Significantly Inhibited Intestinal Permeability of IECs

**Fig. 1:**
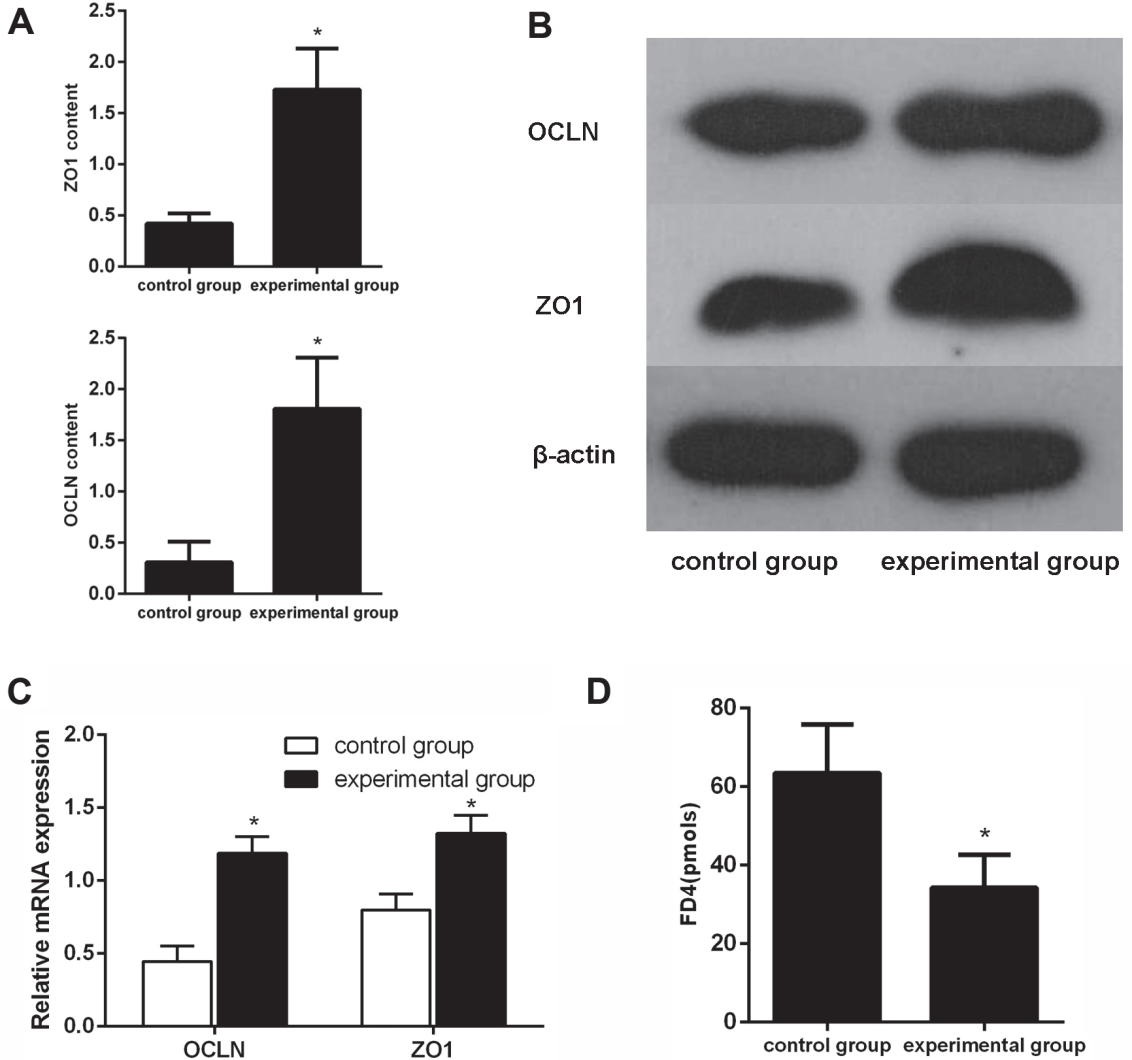
*L. casei* LC01 significantly inhibited intestinal permeability of IECs. (**A**) ELISA showed that the expression levels of OCLN and ZO1 were significantly increased in IECs under *L. casei* LC01 treatment compared to IECs that did not receive *L. casei* LC01 treatment for 3 days. (B & C) Protein and mRNA expression levels of OCLN and ZO1 were significantly upregulated in the experimental group compared with the control group. (D) FD4 was significantly downregulated in the experimental group compared with the control group. *p < 0.05.

IECs treated with or without *L. casei* LC01 for 3 days were considered the experimental and control group, respectively. As detected by ELISA, OCLN and ZO1 were significantly more highly expressed in IECs under *L. casei* LC01 treatment when compared to IECs that did not receive *L. casei* LC01 treatment (Fig. 1A). Further examination revealed that the protein and mRNA expression levels of OCLN and ZO1 were significantly upregulated in the experimental group compared with the control group (Figs. 1B and 1C). FD4 was significantly downregulated in the experimental group compared with the control group (Fig. 1D). These results strongly suggested that *L. casei* LC01 significantly inhibited intestinal permeability of IECs.

### *L. casei* LC01 Suppressed the Expression of miR-144 in IECs

**Fig. 2:**
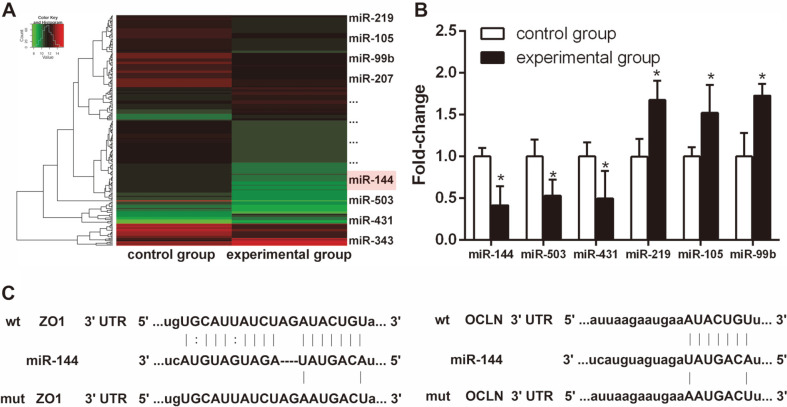
*L. casei* LC01 decreased the expression of miR-144 in IECs. (**A**) Heatmap showing that 26 miRNAs were differentially expressed in IECs under *L. casei* LC01 treatment, of which 10 were upregulated and 16 were downregulated. (**B**) Six miRNAs (including miR-144) were randomly selected to verify the reliability of the microarray analysis through qRT-PCR.(**C**) OCLN and ZO1 share a matching 3′ UTR sequence that targets the seed region of miR-144. *p < 0.05 (Tukey’s test).

To study the differential expression of miRNAs in IECs in response to *L. casei* LC01, we treated IECs with *L. casei* LC01 for 3 days. IECs treated with or without *L. casei* LC01 were subjected to microarray analysis to identify differentially expressed miRNAs. The results showed that miR-144 was significantly downregulated in IECs during *L. casei* LC01 treatment (Fig. 2A), which is similar to our previous report [14]. To further verify its reliability, 6 miRNAs (including miR-144) were randomly selected for qRT-PCR. Consistent with the previous findings, the expression of miR-144 was significantly downregulated in IECs treated with *L. casei* LC01 compared to the *L. casei* LC01-untreated group (Fig. 2B). Bioinformatics analyses and our previous study[14] found that OCLN and ZO1 mRNAs contain a matching 3’UTR sequence that targets the seed region of miR-144, suggesting that OCLN and ZO1 are potential target genes of miR-144 (Fig. 2C).

### *L. casei* LC01 Regulated Intestinal Permeability of IECs Via miR-144

**Fig. 3:**
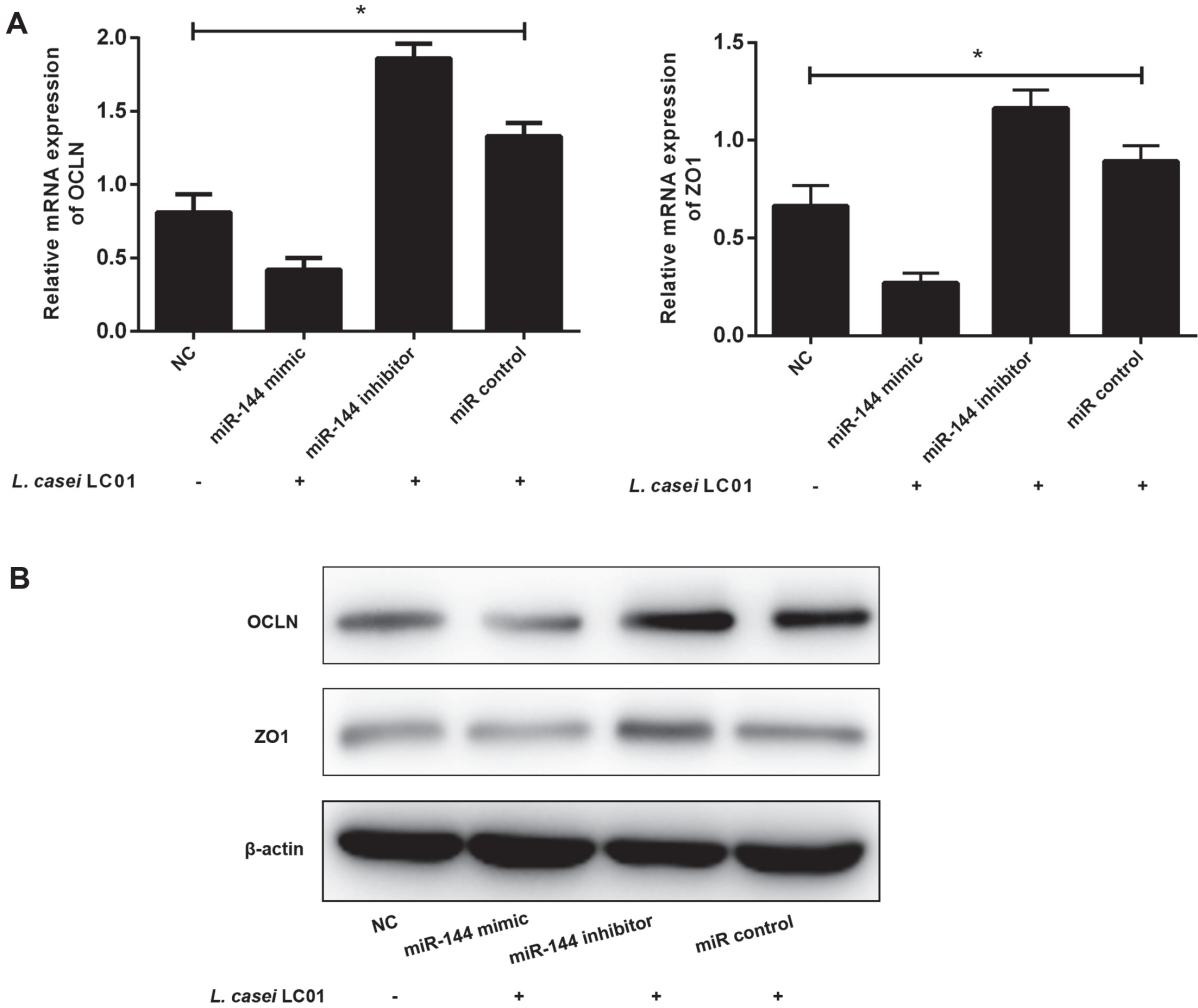
*L. casei* LC01 regulated intestinal permeability of IECs via miR-144. (**A**) qRT-PCR results showed that the expression levels of OCLN, ZO1 were significantly decreased in the miR-144 mimic group but were enhanced in the miR-144 inhibitor and miR control group compared with NC group. (**B**) The western blot results were consistent with the qRT-PCR results. *p < 0.05, difference between all groups (ANOVA & LSD).

To evaluate the role of miR-144 in IECs during *L. casei* LC01 treatment, we transfected IECs with RNA oligoribonucleotides under *L. casei* LC01 treatment for 3 days and then tested gene expression by qRT-PCR and western blot. OCLN and ZO1 were significantly decreased in the miR-144 mimic group but were enhanced in the miR-144 inhibitor and miR control group compared with NC group, as shown by qRT-PCR (Fig. 3A). Western blot results also demonstrated that the changes in protein levels were similar to those in mRNA levels (Fig. 3B). These data suggest that *L. casei* LC01 regulates intestinal permeability of IECs via miR-144.

### miR-144 Promoted Intestinal Permeability of IECs More Strongly than si-OCLN and si-ZO1 during *L. casei* LC01 Treatment

**Fig. 4:**
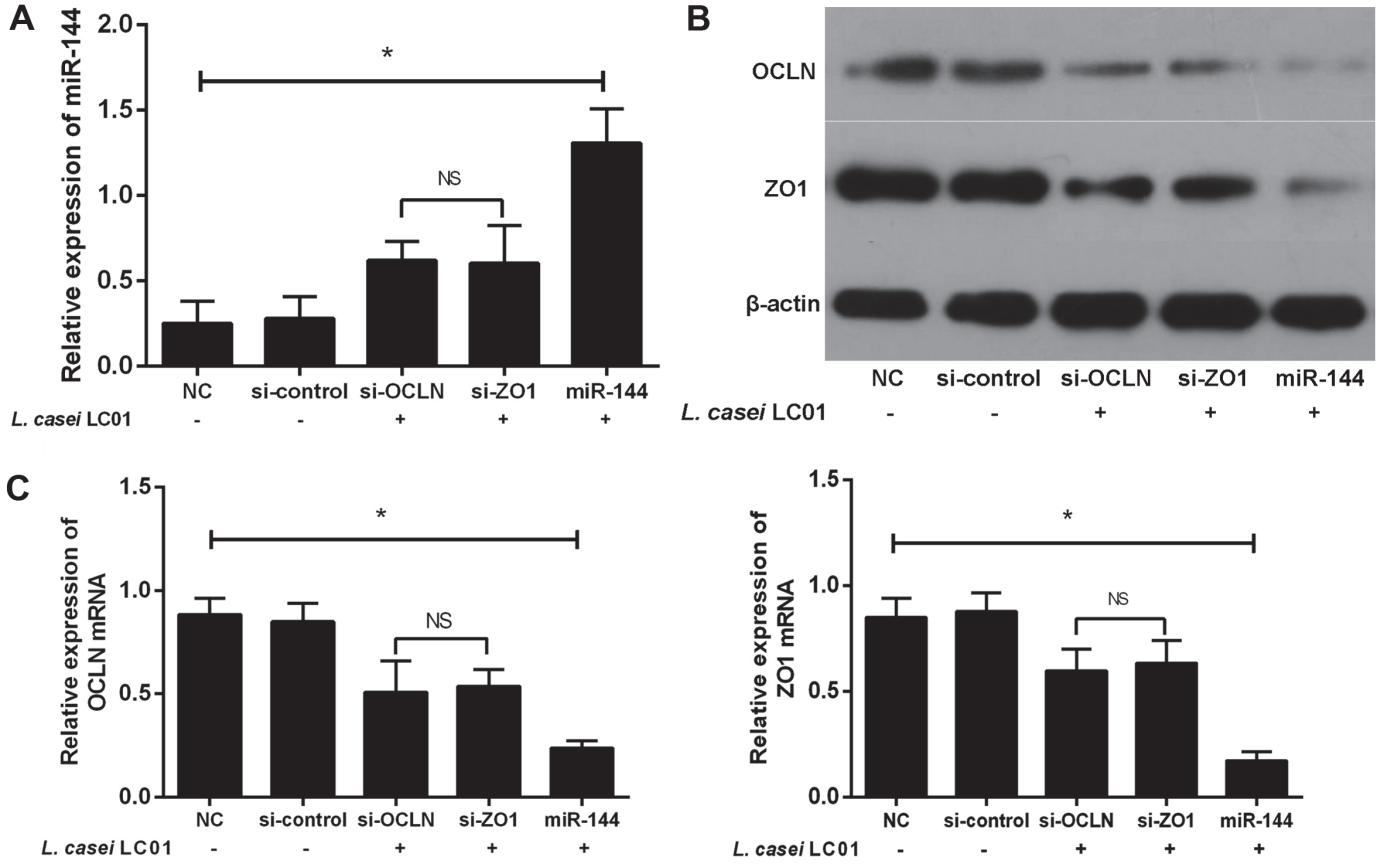
miR-144 strongly promoted intestinal permeability of IECs compared to si-OCLN and si-ZO1 during *L. casei* LC01 treatment. (**A**) qRT-PCR demonstrated successful transfection of miR-144, si-OCLN and si-ZO1. (B and C) Western blot and qRT-PCR results both showed that OCLN and ZO1 were significantly decreased in the miR-144, si-OCLN, and si-ZO1 groups compared to the NC and si-control group. In addition, OCLN and ZO1 were the most decreased in the miR-144 group compared with all other groups. *p < 0.05, difference between all groups; ^NS^*p* > 0.05, difference between the si-OCLN and si-ZO1 groups (ANOVA & LSD).

First, we transfected IECs with miR-144, si-OCLN or si-ZO1, and verified the transfection efficiency of miR-144, si-OCLN, and si-ZO1 in IECs by qRT-PCR (Fig. 4A). Next, we examined the expression of OCLN and ZO1 by western blot (Fig. 4B) and qRT-PCR (Fig. 4C) during *L. casei* LC01 treatment. The results demonstrated that OCLN and ZO1 were significantly downregulated in the miR-144, si-OCLN, and si-ZO1 groups compared with the NC group and si-control group, of which expression in the miR-144 group was reduced the most. These results suggested that miR-144 promoted intestinal permeability of IECs more strongly than si-OCLN and si-ZO1 during *L. casei* LC01 treatment.

### Overexpression of OCLN/ZO1 Partially Rescued the Promoting Effect of miR-144 on Intestinal Permeability in IECs during *L. casei* LC01 Treatment

**Fig. 5:**
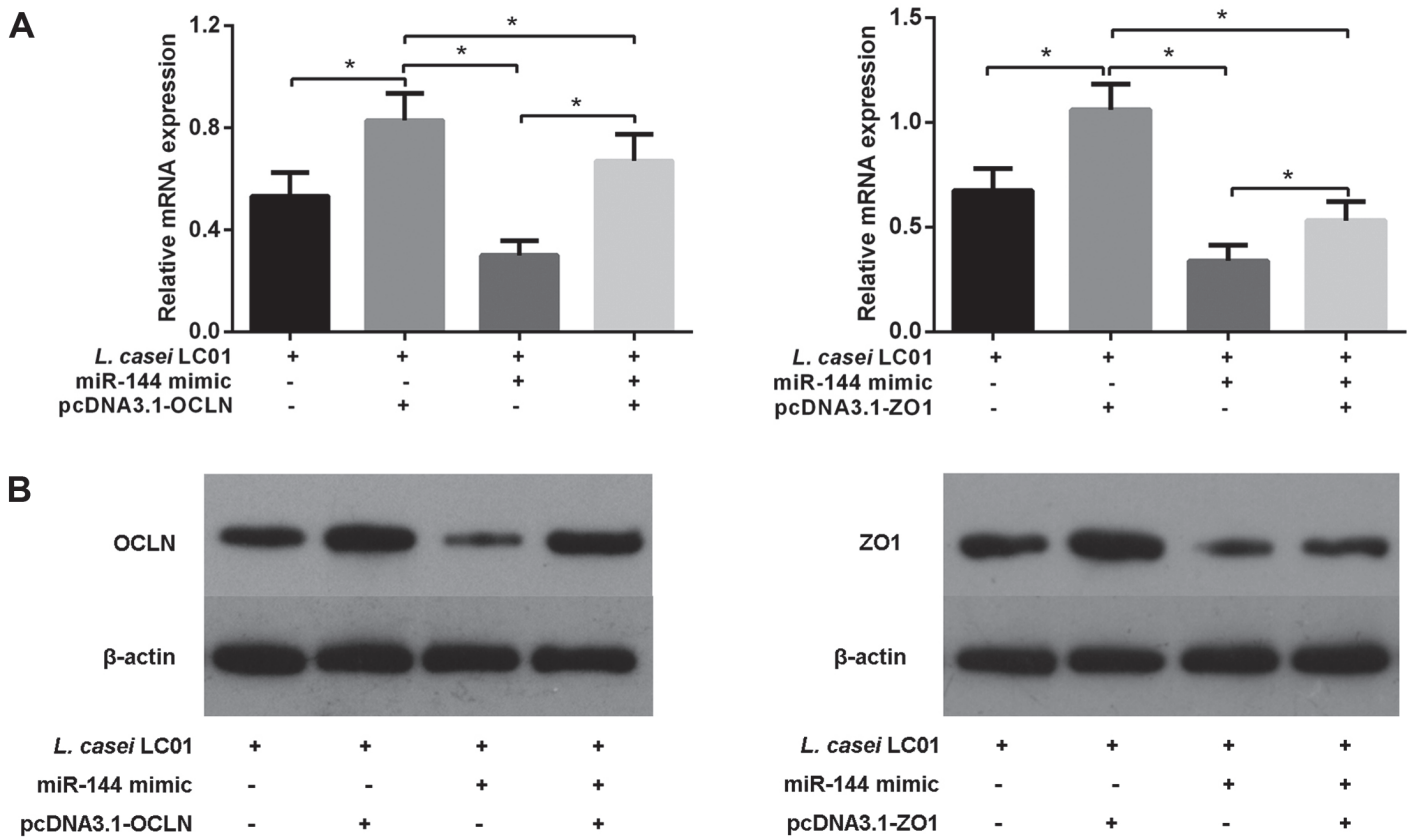
Overexpression of OCLN/ZO1 partially rescued the promoting effect of miR-144 on intestinal permeability in IECs during *L. casei* LC01 treatment. (**A**) qRT-PCR showed that pcDNA3.1-OCLN/ZO1 remarkably increased the expression of OCLN/ZO1, but miR-144 suppressed OCLN/ZO1 expression in IECs during *L. casei* LC01 treatment. Moreover, pcDNA3.1-OCLN/ZO1 also markedly increased OCLN/ZO1 expression, even in the presence of miR-144. (**B**) The western blot results were consistent with the qRT-PCR results.

To further understand the interaction between miR-144, OCLN/ZO1 and *L. casei* LC01 on regulating intestinal permeability of IECs, we transfected IECs with miR-144 and pcDNA3.1-OCLN/ZO1 under *L. casei* LC01 treatment. We observed that miR-144 suppressed OCLN/ZO1 expression, and pcDNA3.1-OCLN/ZO1 remarkably increased the expression of OCLN/ZO1 in IECs during *L. casei* LC01 induction. Moreover, pcDNA3.1-OCLN/ZO1 also markedly increased OCLN/ZO1 expression even in the presence of miR-144 (Figs. 5A and 5B). In other words, pcDNA3.1-OCLN/ZO1 partially rescued the effect of miR-144 on IECs under *L. casei* LC01 treatment, restoring the expression of OCLN/ZO1. These results indicated that overexpression of OCLN/ZO1 partially rescued the promoting effect of miR-144 on intestinal permeability in IECs during *L. casei* LC01 treatment.

## Discussion

In a previous study, intestinal microflora were reported to play an important role in the maintenance of the host intestinal epithelial barrier function [5]. Some probiotics can act through molecular and cellular mechanisms that prevent pathogenic bacterial adhesion, enhance innate immunity, decrease pathogen-induced inflammation and promote intestinal epithelial cell survival, barrier function, and protective responses [19]. It has been reported that *L. casei* DG can help regulate intestinal barrier function, which may be related to miRNA-mediated regulation of mRNA [18]. In our previous report [17], we confirmed that miR-144 was upregulated in IBS-D rats, which showed a promoting effect on intestinal epithelial permeability and may be a key regulator of intestinal epithelial permeability in IBS-D. However, the exact role of miR-144 in the development of IBS-D and how gut microflora participate in this process remain largely unclear.

The aim of this study was to further identify the molecular mechanisms underlying the mutual effect of *L. casei* LC01, miR-144 and its target genes on the regulation of the intestinal permeability of IECs. The major findings of the present study were as follows: 1) *L. casei* LC01 suppressed the expression of miR-144 and FD4 and promoted OCLN and ZO1 expression in IECs; 2) *L. casei* LC01 enhanced the barrier function in IECs via downregulation of miR-144 and upregulation of OCLN and ZO1; 3) Under *L. casei* LC01 treatment, OCLN and ZO1 overexpression could partially eliminate the promoting effect of miR-144 on intestinal permeability in IECs. These findings suggested that *L. casei* LC01 regulates intestinal permeability in IECs via miR-144 targeting of OCLN/ZO1, which might be a potential strategy for managing or preventing epithelial barrier dysfunction.

Recent evidence indicates that the communication between the gut microbiome and the host may be partially carried out by miRNAs, which are differentially expressed under specific microflora states [20]. However, the role of miRNAs in the microbial-host communication of intestinal diseases is not yet fully understood. Interestingly, an important finding of the present study is that miR-144 was downregulated in IECs during *L. casei* LC01 treatment, similar to previous reports [21, 22], while in our own previous work we confirmed that miR-144 plays vital roles in regulating intestinal epithelial permeability in IBS-D rats [17], suggesting that *L. casei* LC01 could regulate intestinal permeability of IECs via miR-144.

IECs are key mediators of intestinal homeostasis and are able to establish an immune environment that allows symbiotic bacterial colonization. IECs provide physical and biochemical barriers that sequester host tissues and commensal bacteria to maintain intestinal homeostasis. IECs have a mechanism enabling them to respond to microbial signals, which confers tolerance against continuous exposure to symbiotic bacteria [23]. In this study, IECs were used to investigate intestinal epithelial permeability stimulated by *L. casei* LC01. Both OCLN and ZO1 are potential direct target genes of miR-144 [17], which are tight junction proteins closely related to intestinal epithelial permeability and intestinal epithelial barrier function [24]. ELISA, qRT-PCR and western blot results showed that the expression levels of OCLN and ZO1 were significantly increased in IECs under *L. casei* LC01 treatment when compared to untreated IECs. These results suggest that *L. casei* LC01 could enhance intestinal epithelial barrier function in IECs via downregulation of miR-144 and upregulation of OCLN and ZO1.

It has also been reported that OCLN and ZO1 are not only capable of controlling paracellular macromolecule permeability but also interacting with intracellular signaling pathways that regulate and maintain intestinal epithelial permeability, subsequently enhancing intestinal epithelial barrier function [25-29]. To further understand the interaction between miR-144, OCLN/ZO1 and *L. casei* LC01 on regulating intestinal permeability in IECs, we transfected IECs with miR-144 and pcDNA3.1-OCLN/ZO1 under *L. casei* LC01 treatment. As a result, OCLN/ZO1 levels were significantly decreased in the miR-144 and si-OCLN/ZO1 groups compared with the control group and model group, of which expression in the miR-144 group was reduced the most. In other words, miR-144 upregulation showed a stronger effect on attenuating epithelial barrier function and promoting intestinal permeability in IECs than si-OCLN and si-ZO1 did under *L. casei* LC01 treatment. Furthermore, overexpression of OCLN/ZO1 partially rescued the promoting effect of miR-144 on intestinal permeability in IECs under *L. casei* LC01 treatment, indicating that *L. casei* LC01 regulates intestinal permeability in IECs via miR-144 targeting of OCLN/ZO1. The above results suggest that targeted microbial interventions may help restore or control intestinal epithelial permeability.

In addition, the results of this study have several important clinical implications. First, we conducted miRNA microarray analysis in this study, which provides examples of identifying differentially expressed miRNAs and their target genes after intervention with beneficial bacteria. Second, we provide the basis for further research and characterization of the beneficial bacteria-miRNA-OCLN/ZO1 relationship, which not only helps to better understand the key molecular switches regarding the permeability of the intestinal mucosa but also to characterize the miRNAs related to intestinal epithelial permeability-related diseases. Third, *L. casei* LC01 could be used together with miR-144 as a therapeutic strategy to promote mucosal barrier function and become a new target for the treatment of intestinal epithelial barrier dysfunction-related diseases.

In summary, regulation of intestinal epithelial permeability through tight junction proteins is an important mechanism for enhancing mucosal defense functions and maintaining intestinal homeostasis. *L. casei* LC01 intervention can inhibit miR-144 expression in IECs and target OCLN and ZO1 expression to maintain intestinal homeostasis. Regulation of intestinal epithelial permeability through probiotics may become a new target for the treatment of intestinal epithelial barrier dysfunction-related diseases.
